# Tracheostomy management in patients with severe acute respiratory distress syndrome receiving extracorporeal membrane oxygenation: an International Multicenter Retrospective Study

**DOI:** 10.1186/s13054-021-03649-8

**Published:** 2021-07-07

**Authors:** Matthieu Schmidt, Christoph Fisser, Gennaro Martucci, Darryl Abrams, Thomas Frapard, Konstantin Popugaev, Antonio Arcadipane, Bianca Bromberger, Giovanni Lino, Alexis Serra, Sacha Rozencwajg, Matthias Lubnow, Sergey Petrikov, Thomas Mueller, Alain Combes, Tài Pham, Daniel Brodie, for the International ECMO Network (ECMONet)

**Affiliations:** 1 Sorbonne Université, Paris 06, INSERM UMRS_1166-iCAN, Institute of Cardiometabolism and Nutrition, 75651 Paris Cedex 13, France; 2 Assistance Publique–Hôpitaux de Paris, Pitié–Salpêtrière Hospital, Medical Intensive Care Unit, 47, bd de l’Hôpital, 75651 Paris Cedex 13, France; 3 Department of Internal Medicine II, University Hospital Regensburg, Regensburg, Germany; 4 IRCCS-ISMETT Instituto Mediterraneo per i Trapianti e Terapie ad alta specializzazione - Department of Anesthesia and Intensive Care, Palermo, Italy; 5 Department of Medicine, Columbia University College of Physicians & Surgeons, New York, NY USA; 6 Center for Acute Respiratory Failure, New York-Presbyterian Hospital, New York, NY USA; 7 Sklifosovsky Research Institute of Emergency Medicine, Bolshaya Sukharevskaya squire, 3, Moscow, Russia; 8 Université Paris-Saclay, AP-HP, Service de médecine intensive-réanimation, Hôpital de Bicêtre, DMU CORREVE, FHU SEPSIS, Groupe de Recherche Clinique CARMAS, Le Kremlin-Bicêtre, France; 9 Université Paris-Saclay, UVSQ, Univ. Paris-Sud, Inserm, Equipe d’Epidémiologie respiratoire intégrative, CESP, Villejuif, France

**Keywords:** ECMO, Acute respiratory distress syndrome, Mechanical ventilation, Tracheostomy, Bleeding, Outcome

## Abstract

**Background:**

Current practices regarding tracheostomy in patients treated with extracorporeal membrane oxygenation (ECMO) for acute respiratory distress syndrome are unknown. Our objectives were to assess the prevalence and the association between the timing of tracheostomy (during or after ECMO weaning) and related complications, sedative, and analgesic use.

**Methods:**

International, multicenter, retrospective study in four large volume ECMO centers during a 9-year period.

**Results:**

Of the 1,168 patients treated with ECMO for severe ARDS (age 48 ± 16 years, 76% male, SAPS II score 51 ± 18) during the enrollment period, 353 (30%) and 177 (15%) underwent tracheostomy placement during or after ECMO, respectively. Severe complications were uncommon in both groups. Local bleeding within 24 h of tracheostomy was four times more frequent during ECMO (25 vs 7% after ECMO, *p* < 0.01). Cumulative sedative consumption decreased more rapidly after the procedure with sedative doses almost negligible 48–72 h later, when tracheostomy was performed after ECMO decannulation (*p* < 0.01). A significantly increased level of consciousness was observed within 72 h after tracheostomy in the “after ECMO” group, whereas it was unchanged in the “during-ECMO” group.

**Conclusion:**

In contrast to patients undergoing tracheostomy after ECMO decannulation, tracheostomy during ECMO was neither associated with a decrease in sedation and analgesia levels nor with an increase in the level of consciousness. This finding together with a higher risk of local bleeding in the days following the procedure reinforces the need for a case-by-case discussion on the balance between risks and benefits of tracheotomy when performed during ECMO.

**Supplementary Information:**

The online version contains supplementary material available at 10.1186/s13054-021-03649-8.

## Introduction

Venovenous extracorporeal membrane oxygenation (VV-ECMO) in adults with severe acute respiratory distress syndrome (ARDS) has been shown to be associated with better outcomes than conventional mechanical ventilation alone in the extracorporeal membrane oxygenation to rescue acute lung injury in severe acute respiratory distress syndrome (EOLIA) trial [1], with that position supported by the post hoc Bayesian analysis of EOLIA [2], and 2 subsequent meta-analyses [3, 4]. With the ultimate goal of using ECMO to protect the lung by minimizing ventilator-induced lung injury beyond current standard of care [5], an ultra-protective lung ventilation strategy targeting very low driving pressures and tidal volumes has largely been adopted across centers with high ECMO volume [6]. However, these patients are likely to receive prolonged mechanical ventilation. For instance, mechanical ventilation duration was 22 (11–34) days in the ECMO arm of the EOLIA trial [1], whereas it was 40 (23–68) days in a non-selected group of 84 ECMO patients surviving to 6 months [7]. Tracheostomy is generally a common procedure for patients who require prolonged mechanical ventilation. Recent trials comparing “early” tracheostomy (i.e., within 8 days of endotracheal intubation) versus “late” tracheostomy (i.e., after at least 10 days of mechanical ventilation) in non-ECMO patients [8, 9] found no difference in overall mortality, hospital or intensive care unit (ICU) length of stay. Tracheostomy may be beneficial by lowering airway resistance, improving oral hygiene, and making the airway more secure [10]. It is also associated with less sedative and analgesic administration, earlier oral nutrition, better comfort, and ease of care [8, 11]. However, these benefits were reported in a general critically ill population. The risks and outcomes may differ when applied to patients receiving ECMO, given the severity of illness, ECMO-induced coagulopathy, frequent thrombocytopenia, and high risk of bleeding in these patients, which could influence the decision for tracheostomy, adverse event profiles, and outcomes. Currently available data in this specific population are scarce and limited to small, single-center cohorts [12–14].

The objectives of this international, multicenter, retrospective study were: (1) to assess the prevalence, timing, and tracheostomy-related management of ECMO-supported ARDS patients in large volume ECMO centers; (2) to investigate the association between the timing of the tracheostomy (i.e., during or after ECMO) and related complications, sedative, analgesic, and transfusion use; and (3) to report factors influencing the decision to perform a tracheostomy during ECMO or after ECMO decannulation in that population.

## Methods

### Study design, patients

This study included all consecutive adult patients with ECMO-supported severe ARDS hospitalized in four international ICUs with a high volume of ECMO cases annually (> 30 ECMO runs/year) [15] between January 2009 and December 2017. Patients undergoing extracorporeal CO_2_ removal, those with end-stage chronic respiratory failure, younger than 15 years old, or with a tracheostomy before ECMO initiation were excluded from the final analysis. All participating ICUs obtained institutional review board approval by following their local regulations (Additional file 1).

### Tracheostomy procedure during ECMO

All centers had a tracheostomy procedure, which was similarly performed with or without ECMO (see Additional file 1). Heparin was stopped for four hours pre-procedure and usually restarted approximately 2 h post-procedure in the absence of significant local bleeding. Minimum platelet count and fibrinogen levels considered acceptable for the procedure ranged from 50,000 to 80,000 G/L, and 150 to 200 mg/dL, respectively.

All patients were managed with goal-directed sedation, guided by the Richmond Agitation Sedation Scale (RASS) (26), which was monitored and evaluated by nurses 2 to 6 times daily, depending on the center. ICU nurse-to-ECMO patient(s) ratio ranged from 1:1 to 1:2 (Additional file 2).

### Data collection

Data collection is detailed in the online supplement. Chronic respiratory disease included asthma, chronic interstitial lung disease, chronic obstructive lung disease, chronic restrictive lung disease, and/or obstructive sleep apnea. Immunocompromised status was defined as hematological malignancies, an active solid tumor or having received specific anti-tumor treatment within 1 year, solid-organ transplant, human immunodeficiency virus-infected, or long-term corticosteroids or immunosuppressants. Major bleeding was defined as requiring two or more units of packed red blood cells within 24 h due to an obvious hemorrhagic event, or necessitating a surgical or interventional procedure, or an intracerebral hemorrhage, or causing a fatal outcome [16].

The time between ECMO and tracheostomy was collected, and patients were classified as tracheostomy performed “during” or “after ECMO”. Any side effects or technical problems that occurred within 24 h after the procedure (i.e., early complications), such as local bleeding, pneumothorax, subcutaneous emphysema, tracheal rupture, or tracheostomy failure were recorded. Similarly, late complications including local bleeding (i.e., requirement of at least one red blood cell transfusion and/or surgical intervention), accidental tracheostomy decannulation, and tracheomalacia were collected. RASS, cumulative consumption of propofol, midazolam, analgesics (expressed as sufentanil-equivalent doses), and transfusion products were collected 48 h before and after tracheostomy. Similarly, the daily heparin dose was reported 24 h before and after the procedure, whereas “the first day awake” was defined as the first day where the RASS score was ≥ 0 for more than 12 h.

Lastly, patient outcomes included the date of ECMO decannulation, date of liberation from mechanical ventilation, and vital status at hospital discharge.

### Statistical analyses

This study followed the Consolidated Standards of Reporting Trials (CONSORT) recommendations for reporting cohort studies (STROBE statement) [17]. Normal distribution was tested using a Shapiro–Wilk test. Continuous variables (expressed as median [25th–75th percentile] or mean ± standard deviation) were compared with the student’s t-test, or the Kruskal–Wallis test, as appropriate. In addition, when there were more than 2 groups (“during ECMO tracheostomy”, “after ECMO tracheostomy”, and “no tracheostomy”), pairwise comparisons adjusting for multiple testing (Tukey or Benjamini and Hochberg methods) were performed.

Pre-ECMO factors associated with tracheostomy were assessed within the whole cohort using univariate and multivariate logistic regression models. Pre-ECMO variables (i.e., obtained within 24 h before ECMO cannulation) included in the models were defined a priori, and no variable selection was performed. Therefore, the following variables were included: age, body mass index, center, immunocompromised status, SOFA score without respiratory and neurological components, the time between mechanical ventilation and ECMO, surgery within 7 days before ECMO, prone positioning, pneumothorax, corticosteroids, cardiac arrest before ECMO, extra pulmonary infection, known restrictive lung disease, and bacterial or viral pneumonia, or pancreatitis. Because the neurological SOFA component was not defined similarly between centers (i.e., based on the Glasgow scale before intubation or a 3 or 4 for a patient already sedated) it was not retained in the model. Similarly, a pulmonary SOFA component of 4 was expected in all of these severe ARDS patients before ECMO. Multiple imputations were used to replace missing values where appropriate. Odd ratios and their 95% confidence intervals were estimated. A *p* value < 0.05 was considered statistically significant. Statistical analyses were conducted with R v4.0.1.

## Results

### Participating ICUs and patients enrolled

**Fig. 1:**
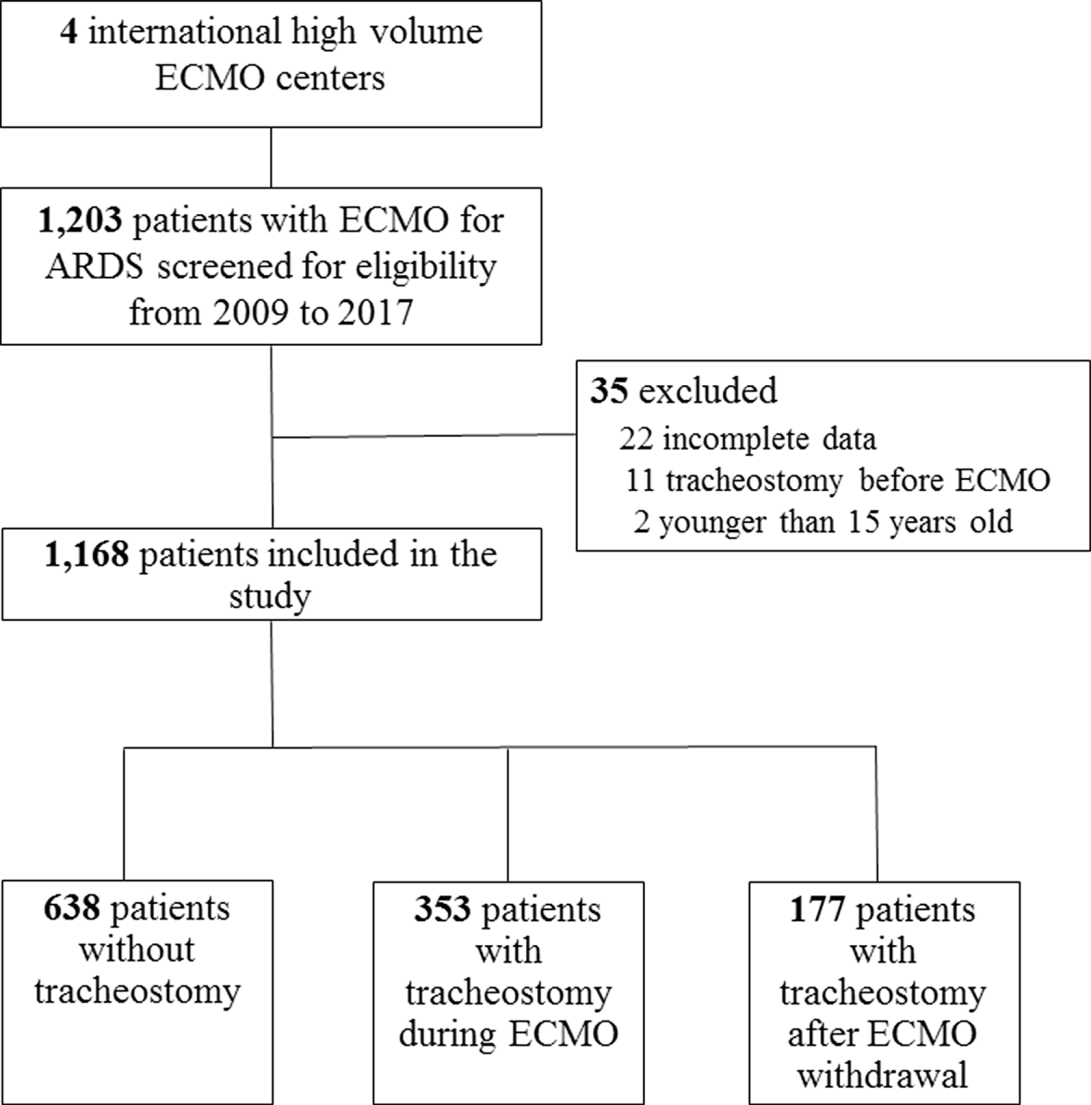
Flow Chart of the study. ARDS, acute respiratory distress syndrome; ECMO, extracorporeal membrane oxygenation

Four international ECMO centers from Italy, Germany, USA, and France which treated from 57 to 305 patients with VV or venoarterial ECMO during the previous calendar year, participated in the study. 1,168 patients were treated with VV-ECMO during the study period. The main characteristics of the centers are described in Online Table 1. Of the 1,168 patients treated with VV-ECMO for severe ARDS (age 48 ± 16 years, 76% male, SAPS II score 51 ± 18) during the enrollment period, 353 (30%) and 177 (15%) underwent tracheostomy during ECMO or after ECMO decannulation, respectively (Fig. 1). Their characteristics at ECMO initiation and their outcomes are reported in Table 1. Median (interquartile range) time between endotracheal intubation and ECMO initiation was 2 (1–6) days. Those who never received a tracheostomy were sicker, with a significantly higher SAPS II score, SOFA score, lactate at cannulation, and more frequently suffered pre-ECMO cardiac arrest than those who received a tracheostomy. Chronic respiratory disease and an immunocompromised status were more frequent in tracheostomized patients than in those who did not receive a tracheostomy (*p* < 0.001). Noticeably, pre-ECMO ventilation parameters and arterial blood gases, including the ratio of the partial pressure of arterial oxygenation to the fraction of inspired oxygen were similar in both groups.

**Table 1:** Baseline characteristics and outcomes according to the timing of tracheostomy

	All patients (*n* = 1168)	During ECMO tracheostomy (*n* = 353)	After ECMO tracheostomy (*n* = 177)	No tracheostomy (*n* = 638)	*P*
Age, years	48 ± 16	48 ± 15	48 ± 15	48 ± 16	0.77
Male sex	894 (76)	274 (78)	129 (73)	491 (77)	0.48
Body mass index, kg/m^2^	30 ± 9	30 ± 9	31 ± 8	30 ± 9	0.20
Chronic respiratory disease ^a^	162 (14)	90 (25)	34 (19)	38 (6)^#*^	< 0.01
Immunodeficiency	177 (15)	77 (22)	34 (19)	66 (10)^#*^	< 0.01
SAPS II score	51 ± 18	50 ± 16	47 ± 17	53 ± 20^*^	0.01
SOFA at cannulation	10.5 ± 3.2	9.9 ± 2.8	10.1 ± 3.1	10.8 ± 3.4^#*^	< 0.01
RESP score	3.2 ± 2.9	2.8 ± 3.0	3.6 ± 2.8	3.2 ± 2.9	0.02
ARDS risk factor					< 0.01
Bacterial pneumonia	564 (48)	177 (50)	68 (38)	319 (50)	
Viral pneumonia	195 (17)	69 (19)	49 (28)	77 (12)	
Pancreatitis	10 (1)	1 (0)	6 (3)	3 (0.5)	
Others	399 (44)	106 (31)	54 (31)	239 (38)	
Post-operative ARDS (< 7 days)	245 (21)	77 (22)	61 (34)^#^	107 (17) ^*^	< 0.01
Pre-ECMO ventilatory mechanic					
Respiratory rate, breaths/min	25 ± 7	26 ± 7	25 ± 8	25 ± 7	0.02
Static compliance, ml/cmH_2_O	26 ± 12	25 ± 12	26 ± 12	26 ± 12	0.22
Pre-ECMO cardiac arrest	153 (13)	36 (10)	21 (12)	96 (15)	0.09
Pre-ECMO pneumothorax	96 (8)	49 (14)	26 (14)	21 (3)^#*^	< 0.01
Interval MV–ECMO, days	2 (1–6)	3 (1–7)	1 (1–4)	2 (1–6)	< 0.01
Venovenous-ECMO	1165 (100)	352 (100)	176(100)	637 (100)	0.38
Femoral–jugular	997 (85)	293 (83)	151 (85)	553 (87)	
Femoral–femoral	50 (4)	17 (5)	12 (7)	21 (3)	
Dual lumen cannula	121 (10)	43 (12)	14 (8)	64 (10)	
Pre-ECMO blood gases					
pH	7.23 ± 0.14	7.24 ± 0.13	7.23 ± 0.15	7.22 ± 0.14	0.16
PaCO_2_, mmHg	66 ± 26	68 ± 29	63 ± 24	65 ± 25	0.15
Arterial lactate, mmol/L	3.7 ± 3.7	2.9 ± 2.9	3.3 ± 3.0	4.1 ± 4.2^#^	< 0.01
PaO_2_/FiO_2_, mmHg	72 ± 37	70 ± 32	70 ± 37	74 ± 40	0.35
Outcomes in ICU					
Renal replacement therapy	461 (39)	177 (50)	99 (56)	185 (29)^#*^	< 0.01
ECMO duration, days	9 (5–16)	19 (11–30)	9 (6–13)^#^	7 (4–11)^#*^	< 0.01
Mechanical ventilation duration, days	20 (11–34)	34 (22–49)	28 (21–38)^#^	13 (7–20)^#*^	< 0.01
ICU LOS, days	25 (14–39)	37 (26–53)	36 (27–47)	16 (10–25)^#*^	< 0.01
Hospital LOS, days	31 (18–49)	44 (31–64)	44 (32–58)	21 (13–35)^#*^	< 0.01
Alive at hospital discharge	746 (64)	238 (67)	154 (87)^#^	354 (55)^#*^	< 0.01

ARDS, acute respiratory distress syndrome; ECMO, extracorporeal membrane oxygenation; ICU, Intensive care unit; LOS, length of stay; MV, mechanical ventilation; PaCO_2_, partial pressure of arterial carbon dioxide; PaO_2_/FiO_2_, ratio of partial pressure of arterial oxygen to fraction of inspired oxygen; RESP, Respiratory ECMO Survival Prediction; SAPS II, simplified acute physiology *score;* SOFA Sequential Organ Failure Assessment

Results are presented as *n* (%), mean ± standard deviation, or median (25th–75th percentiles); ^#^, *p* value < 0.05 vs “tracheostomy during ECMO”; *, *p* value < 0.05 vs “after ECMO tracheostomy”

^a^Including asthma, chronic interstitial lung disease, chronic obstructive lung disease, chronic restrictive lung disease, and obstructive sleep apnea

### Tracheostomy management and complications during or after ECMO

Table 2 compares tracheostomy management and its related complications according to the timing of tracheostomy. In patients who underwent tracheostomy on ECMO, tracheostomy was performed after a median of 8 days on ECMO (range 5–13) (“during-ECMO tracheostomy”), compared to the “after ECMO tracheostomy” group, in which tracheostomy occurred a median of 16 days (range 11–20) after ECMO cannulation. In this group, tracheostomy occurred 6 days (range 4–9) after ECMO decannulation. While the cumulative daily dose of unfractionated heparin received 24 h before the procedure was generally higher in patients tracheostomized during ECMO, unfractionated heparin dose was significantly reduced within the 24 h after the procedure (Additional file 5). The incidence and the severity of early tracheostomy complications were low and similar for the two groups. However, local bleeding was four times more frequent when the tracheostomy was performed during ECMO (25 vs 7% after ECMO, *p* < 0.01). Similarly, major bleeding (apart from the tracheostomy site) was more frequent in the “during ECMO tracheostomy” group than in the “after ECMO tracheostomy” or “no-tracheostomy” groups (31% vs. 14%, *p* < 0.01) (Additional file 3). Overall hospital mortality was 36.1%, with hospital mortality of 32.6%, 13%, and 44.5% in the “during-ECMO tracheostomy”, “after ECMO tracheostomy”, and no-tracheostomy” groups, respectively.

**Table 2:** Tracheostomy management and related complications according to the timing of tracheostomy

	During ECMO tracheostomy (*n* = 353)	After ECMO tracheostomy (*n* = 177)	*P*
Interval ECMO-tracheostomy, days	8 (5–13)	16 (11–20)	< 0.01
Percutaneous dilatational tracheostomy	255 (72)	115 (65)	< 0.01
Mechanical ventilation the day of the tracheostomy			
FiO_2_, %	52 ± 16	44 ± 10	< 0.01
Tidal volume, ml/kg PBW	4.3 ± 2.3	7.5 ± 2.3	< 0.01
Total respiratory rate, breaths/min	20 ± 12	23 ± 9	0.06
Plateau pressure, cmH_2_O	24 ± 4	24 ± 5	0.52
Static compliance, ml/cm H_2_O	23 ± 17	36 ± 16	< 0.01
PEEP, cmH_2_O	11 ± 4	8 ± 3	0.01
Driving pressure, cmH_2_O	14 ± 4	15 ± 4	< 0.01
ECMO settings			
ECMO flow, L/min	3.3 ± 1.3	–	
Sweep gas flow, L/min	5.7 ± 3.0	–	
FdO_2_, %	96 ± 14	–	
PaCO_2_, mmHg	42 ± 8	41 ± 8	0.25
Unfractionated Heparin consumption			
24 h before tracheostomy, UI/24 h	17,280 (8588–39,120)	5192 (0–33,920)	< 0.01
24 h after tracheostomy, UI/24 h	1800 (600–13,000)	500 (0–1500)	< 0.01
Any early tracheostomy complications (first 24 h)	11 (3)	6 (3)	1.00
Local bleeding requiring transfusion	4 (1)	0 (0)	0.30
Pneumothorax	4 (1)	0 (0)	0.30
Subcutaneous emphysema	1 (0.3)	1 (0.5)	1.00
Tracheal rupture	1 (0.3)	2 (1)	0.36
Failure	1 (0.3)	3 (2)	0.18
Any late tracheostomy complications	88 (38)	17 (17)	< 0.01
Local bleeding	87 (25)	13 (7)	< 0.01
Accidental tracheostomy decannulation	1 (0.3)	4 (2)	0.07
Tracheomalacia	0 (0)	1 (1)	–
Delay tracheostomy-awake, days	4 (2–10)	2 (1–4)	< 0.01
Richmond Agitation Sedation Scale			
24 h before the tracheostomy	− 3 (− 4; − 2)	− 3 (− 4; − 1)	< 0.01
24 h after the tracheostomy	− 3 (− 4; − 2)	− 2 (− 3; − 1)	< 0.01

Results are presented as *n* (%), mean ± standard deviation, or median (25th–75th percentiles)

ECMO, extracorporeal membrane oxygenation; FdO_2_, fraction of delivered oxygen; h, hour; MV, mechanical ventilation; PaCO_2_, partial pressure of arterial carbon dioxide; PBW, predicted body weight; PEEP, positive end-expiratory pressure

### Effects of tracheostomy timing

**Fig. 2:**
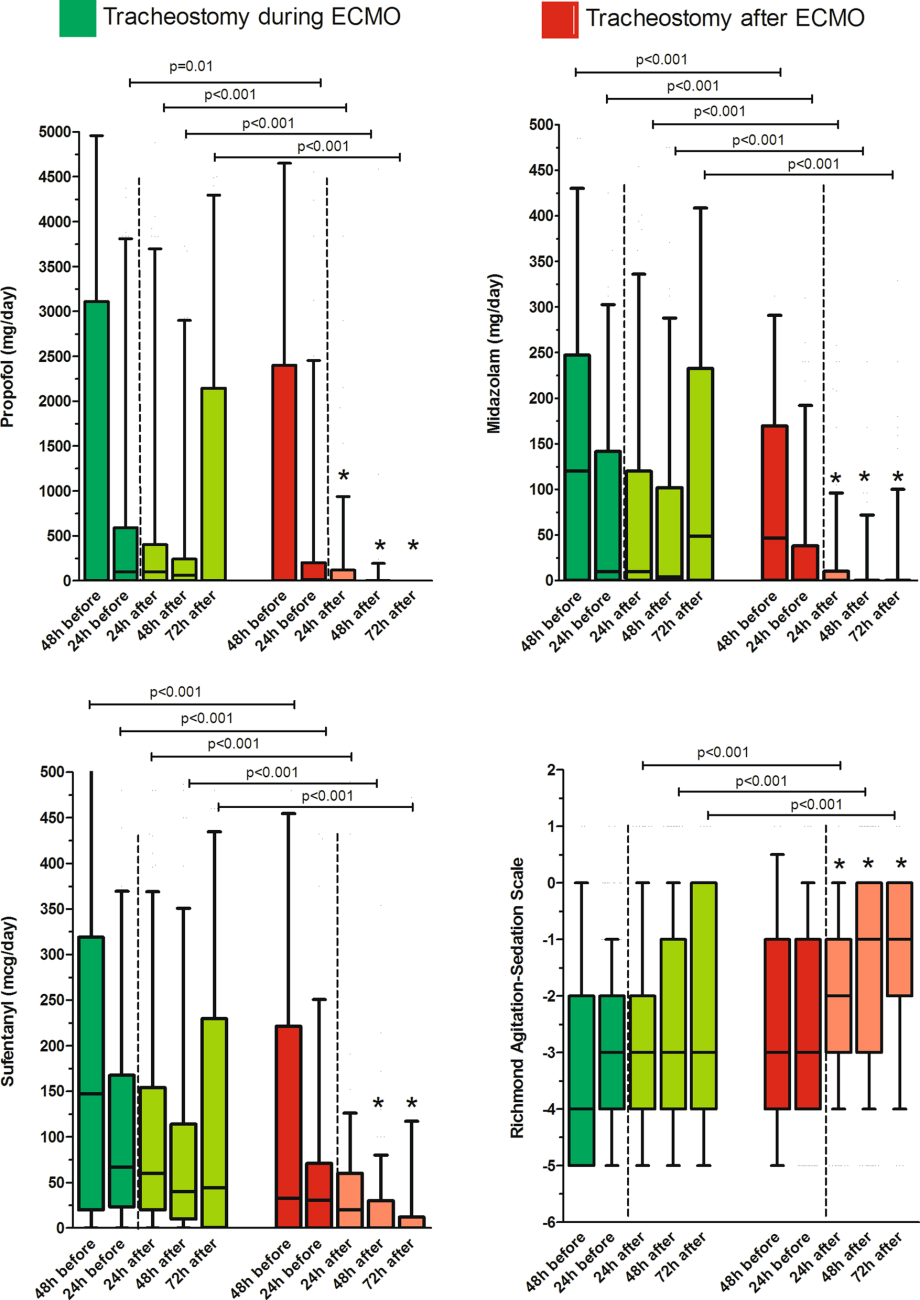
Sedative and analgesic consumption and the Richmond Agitation-Sedation Scale according to the timing of tracheostomy. ECMO, extracorporeal membrane oxygenation; h, hours. *p < 0.05 with 24 h before

Intravenous sedative and analgesic use 48 and 24 h before tracheostomy were consistently lower for after ECMO tracheostomy patients (Fig. 2). Cumulative doses of sufentanil, propofol, and midazolam decreased more rapidly after the procedure for patients tracheostomized after ECMO decannulation. In addition, daily dose of these drugs was significantly lower for the latter group compared to those tracheostomized during ECMO. Propofol and midazolam doses were nearly negligible 48–72 h after the tracheostomy when performed after ECMO decannulation (Fig. 2). In contrast, patients were sedated to similar degrees before and after the procedure in the “during ECMO” group. The level of consciousness was significantly higher before and after the tracheostomy in the “after ECMO” group compared to the “during-ECMO” group. A significant increase of the RASS was observed 24, 48, and 72 h after the tracheostomy in the “after ECMO” group whereas it was unchanged within 72 h after the procedure in the “during-ECMO” group. Similarly, the delay between the tracheostomy and being awake (i.e., RASS score ≥ 0) was significantly longer with the “during ECMO” tracheostomy group (4 [2–10] days vs. 2 [1–4] days, *p* < 0.01). The number of patients who required renal replacement therapy was 50% and 56% in the “during ECMO” and “after ECMO” groups, respectively. Despite similar ICU and hospital lengths of stay in both groups, the survival rate at hospital discharge was significantly greater in the “after ECMO” group (67 vs 87%, *p* < 0.01) (Table 1).

### Pre-ECMO Factors related to the decision to perform a tracheostomy

We observed heterogeneity among high volume ECMO centers in the decision and the timing to perform a tracheostomy, with an increased odds ratio for performing a tracheostomy observed in the centers in New York and Regensburg (Additional file 4). Furthermore, immunocompromised status, pre-ECMO surgery within 7 days, pneumothorax, extrapulmonary infection, prone positioning, and treatment with corticosteroids were significantly associated with the decision to perform a tracheostomy.

The results of the regression model for the tracheostomy procedure during ECMO are reported in Table 3. The time from endotracheal intubation to ECMO initiation had a statistically significant impact, demonstrating a 9% increased chance of tracheostomy during ECMO for each additional day (OR 1.09, 95% CI [1.04–1.15], *p* < 0.01). Similarly, patients receiving corticosteroids before ECMO were more likely to have a tracheostomy on ECMO (OR 1.72 95% CI [1.02–2.96], *p* < 0.01). The model showed that patients in Palermo, those who underwent surgery within 7 days before ARDS, and those suffering from pancreatitis-related ARDS were less likely to be tracheostomized during ECMO. Lastly, patients with higher severity of illness at cannulation had less frequent tracheostomy during ECMO (OR 0.92 95% CI [0.84–1.00], *p* = 0.04) and tracheostomy was, therefore, more likely performed after ECMO decannulation in these patients.

**Table 3:** Pre-ECMO variables associated with the decision to perform a tracheostomy during ECMO (vs. after ECMO) in severe ARDS patients

Variable	OR (95%CI)	*p* value
Pitie Salpetriere hospital, Paris, France	Reference	
Columbia University, New York, United States of America	0.57 (0.28–1.15)	0.12
University Hospital Regensburg, Regensburg, Germany	0.81 (0.43–1.51)	0.51
IRCCS-ISMETT, Palermo, Italy	0.35 (0.14–0.83)	0.02
Age (per year)	0.99 (0.98–1.00)	0.12
Body mass index	1.01 (0.99–1.03)	0.42
Delay from intubation to the initiation of ECMO, for each day	1.09 (1.04–1.15)	< 0.01
Immunocompromised status	1.20 (0.72–2.03)	0.49
Higher SOFA score at cannulation (respiratory and neurological components excluded)	0.92 (0.84–1.00)	0.04
Surgery within 7 days before ARDS onset	0.50 (0.31–0.79)	< 0.01
Prone position before ECMO	0.78 (0.44–1.39)	0.40
Pneumothorax before ECMO	0.89 (0.50–1.61)	0.70
Extra pulmonary infection	1.39 (0.70–2.88)	0.36
Corticosteroids before ECMO	1.72 (1.02–2.96)	0.04
Cardiac arrest before ECMO	0.98 (0.53–1.85)	0.96
Bacterial pneumonia	1.10 (0.67–1.80)	0.70
Viral pneumonia	0.70 (0.38–1.27)	0.24
Pancreatitis	0.09 (0.00–0.56)	0.03

Model performed on 530 patients. Area under the ROC curve of the model 0.75 (0.72–0.78)

ARDS, acute respiratory distress syndrome; CI, confidence interval; ECMO, extracorporeal membrane oxygenation; OR, odds ratio; SOFA Sequential Organ Failure Assessment

## Discussion

To our knowledge, this study is the most extensive assessment of the practice of tracheostomy in patients with ARDS treated with ECMO. The main findings were (1) a tracheostomy was performed in 45% of this large cohort, with 67% of them done during ECMO; (2) tracheostomy appeared safe, even while patients were still receiving ECMO, with uncommon early complications; (3) bleeding around the tracheostomy site was significantly more frequent when the procedure was performed during ECMO than after ECMO decannulation; (4) the reduction in doses of sedative medications and patient awakening occurred later post-tracheostomy when performed during ECMO.

We found that tracheostomy was frequent among ECMO patients in these four high volume ECMO centers. However, the approach to tracheostomy was by no means uniform across ICUs that contributed to this cohort. We found substantial between-center differences in the incidence and timing of tracheostomy, which persisted even after adjustment for pre-ECMO covariates. Percutaneous dilatational tracheostomy was the main technique used during ECMO (76%). Compared to open surgical tracheostomy, this technique (also referred to as bedside tracheostomy), requires a shorter procedure time, eliminates schedule difficulties associated with the operating room, avoids risky transport, and allows pursuing intensive monitoring. Tracheostomy was undertaken in 45% of our study cohort, which is more frequent than in studies in general ARDS cohorts. In a large international, multicenter, unselected, prospective cohort study of patients with ARDS [18], only 14% of the patients with severe ARDS had a tracheostomy [18]. This rate was even lower in the context of COVID-19 with a tracheostomy reported in only 7% of severe ARDS patients [19]. When performed during ECMO, patients had a tracheostomy after 11 (6–20) days of mechanical ventilation, which contrasts with general ARDS patients whose tracheostomy is most commonly performed after 14 days after initiation of mechanical ventilation, and a low proportion of patients received tracheostomy within 7 days [20, 21]. The increased use of tracheostomy in the ECMO population may be attributable to prolonged mechanical ventilation duration and ICU length of stay, a need for airway access for secretion management, and improvement of the patient's comfort while reducing sedation and promoting spontaneous breathing. The optimal timing of tracheostomy has been controversial. Two large randomized controlled trials [8, 9] and a subsequent meta-analysis [22] demonstrated that early tracheostomy (i.e., less than 7 days after intubation) provided no benefit in terms of mechanical ventilation and length of hospital stay, rates of mortality or infectious complications. However, a recent meta-analysis reported lower ventilator-associated pneumonia rates and shorter durations of mechanical ventilation and ICU stay with early tracheostomy [23]. While the number of patients alive at hospital discharge was greater in the after ECMO tracheostomy group compared to the during-ECMO or no tracheostomy groups, competing confounders and an obvious immortality bias preclude assigning any causality between the timing of tracheostomy for ECMO patients and outcomes in our study. Patients with more severe lung impairment, as seen during ECMO, may have a prolonged need for deep sedation, neuromuscular blockade, or prone positioning on ECMO.

The potential expected benefits of tracheostomy in patients who require prolonged ECMO support and mechanical ventilation include less sedation, better comfort, earlier resumption of activity, and prevention of potential vocal cord damage [8, 11]. However, we observed that intravenous sedative and analgesic use decreased more rapidly thereafter for patients with the procedure performed after ECMO decannulation, compared to those receiving tracheostomy during ECMO. Also, sedation and analgesic use and the RASS score did not change within 72 h after the tracheostomy. Still need to suppress the intense respiratory drive, ongoing need for proning or neuromuscular blockade, extremely stiff lungs, and the clinicians’ fear of ECMO cannula dislodgement could be potential explanations [12]. Other additional benefits, which were not investigated in our study, could also include improved patient communication, spontaneous breathing, diaphragm activity, rehabilitation, and participation in early mobilization, as well as earlier oral nutrition [11].

Despite being safe during ECMO with uncommon early complications, we found significantly more frequent local bleeding within 24 h despite the resumption of unfractionated heparin at a lowered dose. Prevention and treatment of hemorrhagic complications are central to the management of patients undergoing ECMO. Alterations in the normal hemostatic balance due to the interaction between patient blood and foreign surfaces, as well as sheer forces from the extracorporeal circuit, combined with acquired coagulation abnormalities and current use of anticoagulation result in high rates of hemorrhagic complications during ECMO support [24]. Considering these complications, it has to be called into question whether it is relevant to perform this procedure during ECMO if not followed by a decrease in the sedation level and an increase of the RASS. Our study reinforces the need to carefully examine the risks and benefits of the need for tracheostomy on a case-by-case basis when performed during ECMO or to reassess whether the goals of performing tracheostomy during ECMO are achievable. An attempt to decrease or wean sedation during ECMO to assess the patient’s tolerance should be considered before performing tracheostomy in that context.

The major strength of this study is the detailed report of the tracheostomy procedure, its early and late complications, and hospital survival status from a large, multicenter series of 1,168 patients with severe ARDS receiving ECMO. We acknowledge several limitations to our study. First, only high-volume ECMO centers participated. They may have specific patient selection criteria and experienced ECMO management that may limit generalizability. Further, when introducing the center variable in the multivariable model, this variable was independently associated with the timing of the tracheostomy, suggesting additional differences in patient characteristics and management may exist across these four high volume centers. Second, our choice to define “the first day awake” as the first day where the RASS score was ≥ 0 for more than 12 h can be called into question, as effective communication with patients could be performed with a RASS score of -2 or -1. Third, variability between clinicians and centers in the timing of tracheostomy insertion and no daily collection of potential cofounders during ECMO [25] make it difficult to draw a true comparison of the outcomes between patients who were tracheostomized during and after ECMO. Also, due to the retrospective design of our study, the reasons that motivate the timing of the procedure were not collected. Our findings should be considered as a first description of the tracheostomy management in experienced ECMO centers. Prospective studies are warranted investigating the timing of tracheostomy and the outcome. Fourth, sedation and analgesic use was only collected within the 3 days following tracheostomy. Longer follow-up may have shown a later decrease in sedation in the “during ECMO” group.

## Conclusion

In conclusion, we found that nearly half of the severe ARDS patients treated with ECMO in high volume centers had a tracheostomy, with two-thirds of them performed during ECMO. This procedure was safe and associated with uncommon early complications even when it was performed during ECMO. Contrary to the patients undertaking tracheostomy after ECMO withdrawal, tracheostomy during ECMO was not associated with an anticipated decrease in sedation and analgesic levels nor an increase of the level of consciousness. This finding and a higher local bleeding risk during the days following the procedure reinforce the need for a case-by-case discussion of the balance between risks and benefits when performing tracheostomy during ECMO. Identifying patients receiving ECMO who are more likely to tolerate a decrease in sedation prior to tracheostomy is warranted. Further studies are now needed to investigate whether early tracheostomy could improve the outcomes of these severely ill patients with expected prolonged mechanical ventilation.

## Supplementary Information

**Additional file 1**. Tracheostomy procedure during ECMO and data collection.

**Additional file 2**. Characteristics of the four international ECMO centers and their tracheostomy management.

**Additional file 3**. ECMO-related complications according to the timing of tracheostomy.

**Additional file 4**. Pre-ECMO variables associated with the decision to perform a tracheostomy (i.e., during or after ECMO) in severe ARDS patients.

**Additional file 5** Impact of tracheostomy on the unfractionated heparin dose and the packed red blood cell transfusion according to the timing of tracheostomy.

## Data Availability

The datasets used during the current study are available from the corresponding author on reasonable request.

## Abbreviations

VV-ECMO: Venovenous extracorporeal membrane oxygenation
ARDS: Acute respiratory distress syndrome
ICU: Intensive care unit
RASS: Richmond Agitation Sedation Scale
CI: Confidence interval

## References

[1] Combes A, Hajage D, Capellier G, Demoule A, Lavoué S, Guervilly C (2018). Extracorporeal membrane oxygenation for severe acute respiratory distress syndrome. N Engl J Med.

[2] Goligher EC, Tomlinson G, Hajage D, Wijeysundera DN, Fan E, Jüni P (2018). Extracorporeal membrane oxygenation for severe acute respiratory distress syndrome and posterior probability of mortality benefit in a post hoc bayesian analysis of a randomized clinical trial. JAMA.

[3] Munshi L, Walkey A, Goligher E, Pham T, Uleryk EM, Fan E (2019). Venovenous extracorporeal membrane oxygenation for acute respiratory distress syndrome: a systematic review and meta-analysis. Lancet Respir Med.

[4] Combes A, Peek GJ, Hajage D, Hardy P, Abrams D, Schmidt M (2020). ECMO for severe ARDS: systematic review and individual patient data meta-analysis. Intensive Care Med.

[5] Rozencwajg S, Guihot A, Franchineau G, Lescroat M, Bréchot N, Hékimian G (2019). Ultra-protective ventilation reduces biotrauma in patients on venovenous extracorporeal membrane oxygenation for severe acute respiratory distress syndrome. Crit Care Med.

[6] Schmidt M, Pham T, Arcadipane A, Agerstrand C, Ohshimo S, Pellegrino V (2019). Mechanical ventilation management during extracorporeal membrane oxygenation for acute respiratory distress syndrome. An International Multicenter Prospective Cohort. Am J Respir Crit Care Med.

[7] Schmidt M, Zogheib E, Roze H, Repesse X, Lebreton G, Luyt CE (2013). The PRESERVE mortality risk score and analysis of long-term outcomes after extracorporeal membrane oxygenation for severe acute respiratory distress syndrome. Intensive Care Med.

[8] Trouillet J-L, Luyt C-E, Guiguet M, Ouattara A, Vaissier E, Makri R (2011). Early percutaneous tracheotomy versus prolonged intubation of mechanically ventilated patients after cardiac surgery: a randomized trial. Ann Intern Med.

[9] Young D, Harrison DA, Cuthbertson BH, Rowan K, TracMan Collaborators (2013). Effect of early vs late tracheostomy placement on survival in patients receiving mechanical ventilation: the TracMan randomized trial. JAMA.

[10] Diehl JL, El Atrous S, Touchard D, Lemaire F, Brochard L (1999). Changes in the work of breathing induced by tracheotomy in ventilator-dependent patients. Am J Respir Crit Care Med.

[11] Nieszkowska A, Combes A, Luyt C-E, Ksibi H, Trouillet J-L, Gibert C (2005). Impact of tracheotomy on sedative administration, sedation level, and comfort of mechanically ventilated intensive care unit patients. Crit Care Med.

[12] Grewal J, Sutt A-L, Cornmell G, Shekar K, Fraser J. Safety and putative benefits of tracheostomy tube placement in patients on extracorporeal membrane oxygenation: a single-center experience. J Intensive Care Med. 2019;885066619837939. 10.1177/0885066619837939 30895877

[13] Kruit N, Valchanov K, Blaudszun G, Fowles J-A, Vuylsteke A (2018). Bleeding complications associated with percutaneous tracheostomy insertion in patients supported with venovenous extracorporeal membrane oxygen support: a 10-year institutional experience. J Cardiothorac Vasc Anesth.

[14] Salna M, Tipograf Y, Liou P, Chicotka S, Biscotti M, Agerstrand C (1992). Tracheostomy is safe during extracorporeal membrane oxygenation support. ASAIO J Am Soc Artif Intern Organs.

[15] Barbaro RP, Odetola FO, Kidwell KM, Paden ML, Bartlett RH, Davis MM (2015). Association of hospital-level volume of extracorporeal membrane oxygenation cases and mortality. Analysis of the extracorporeal life support organization registry. Am J Respir Crit Care Med.

[16] Schulman S, Angerås U, Bergqvist D, Eriksson B, Lassen MR, Fisher W (2010). Definition of major bleeding in clinical investigations of antihemostatic medicinal products in surgical patients. J Thromb Haemost JTH.

[17] STROBE Statement: Home [Internet]. [cited 2020 Aug 21]. Available from: https://www.strobe-statement.org/index.php?id=strobe-home

[18] Bellani G, Laffey JG, Pham T, Fan E, Brochard L, Esteban A (2016). Epidemiology, patterns of care, and mortality for patients with acute respiratory distress syndrome in intensive care units in 50 countries. JAMA.

[19] COVID-ICU Group on behalf of the REVA Network and the COVID-ICU Investigators. Clinical characteristics and day-90 outcomes of 4244 critically ill adults with COVID-19: a prospective cohort study. Intensive Care Med. 2020. 10.1007/s00134-020-06294-x PMC7674575 33211135

[20] Abe T, Madotto F, Pham T, Nagata I, Uchida M, Tamiya N (2018). Epidemiology and patterns of tracheostomy practice in patients with acute respiratory distress syndrome in ICUs across 50 countries. Crit Care Lond Engl.

[21] Mehta AB, Syeda SN, Bajpayee L, Cooke CR, Walkey AJ, Wiener RS (2015). Trends in tracheostomy for mechanically ventilated patients in the United States, 1993–2012. Am J Respir Crit Care Med.

[22] Meng L, Wang C, Li J, Zhang J (2016). Early vs late tracheostomy in critically ill patients: a systematic review and meta-analysis. Clin Respir J.

[23] Chorath K, Hoang A, Rajasekaran K, Moreira A (2021). Association of early vs late tracheostomy placement with pneumonia and ventilator days in critically ill patients: a meta-analysis. JAMA Otolaryngol Head Neck Surg.

[24] Brodie D, Slutsky AS, Combes A (2019). Extracorporeal life support for adults with respiratory failure and related indications: a review. JAMA.

[25] Lederer DJ, Bell SC, Branson RD, Chalmers JD, Marshall R, Maslove DM (2019). Control of confounding and reporting of results in causal inference studies. Guidance for Authors from Editors of Respiratory, Sleep, and Critical Care Journals. Ann Am Thorac Soc.

