# Supplementary material for: Tracheostomy management in patients with severe acute respiratory distress syndrome receiving extracorporeal membrane oxygenation: an International Multicenter Retrospective Study
Source: Crit Care. 2021 Jul 7;25:238. doi: 10.1186/s13054-021-03649-8 (PMC8261805; doi:10.1186/s13054-021-03649-8)
Supplement: Supplementary file 1 — Additional file 1. Tracheostomy procedure during ECMO and data collection. [file 13054_2021_3649_MOESM1_ESM.docx]

**Additional file 1**

**Tracheostomy management in patients with severe acute respiratory distress syndrome receiving extracorporeal membrane oxygenation.**

***An International Multicenter Retrospective Study***

**Tracheostomy procedure during ECMO**

All centers had a tracheostomy procedure, which was similarly performed with or without ECMO (see online supplement). Pre-procedure ultrasound imaging in transverse section was performed to identify vascular structures and reduce the risk of bleeding. In absence of contraindications, percutaneous tracheostomy, performed at the bedside by experienced intensivists or surgeons, was the preferred procedure for all participating centers using the Ciaglia percutaneous technique (Ciaglia Blue Rhino, Cook Critical Care, Bloomington, Illinois). Three centers occasionally used an open surgical technique on ECMO in cases of morbidly obese necks, thyroid enlargement, or diffuse vascularization.

**Data collection**

Age, sex, simplified acute physiology score (SAPS) II (1), Sequential Organ Failure Assessment (SOFA) score (2), immunodeficiency, and risk factors for ARDS were recorded at ICU admission. Respiratory Extracorporeal Membrane Oxygenation Survival Prediction (RESP) score (3) was recorded for the time immediately preceding ECMO implantation. Follow-up variables recorded were renal replacement therapy, major bleeding complications, stroke, red blood cell transfusion, ECMO and mechanical ventilation durations, and lengths of ICU and hospital stay.

**IRB characteristics for each ICUs**

| **ICU name** | **Location** | **IRB number** |
| --- | --- | --- |
| Assistance Publique–Hôpitaux de Paris, Pitié–Salpêtrière Hospital, Medical Intensive Care Unit | Paris, France | National Commission for Informatics and Liberties- no. 2217028v0 |
| Department of Internal Medicine II, University Hospital Regensburg, | Regensburg, Germany | 19-1391-104 - 08.05.2019  Ethic boards, University of Regensburg |
| IRCCS-ISMETT Instituto Mediterraneo per i Trapianti e terapie ad alta specializzazione - Department of Anesthesia and Intensive Care; | Palermo, Italy. | IRRB/34/19 - 27/02/2020 |
| Department of Medicine, Columbia University College of Physicians & Surgeons, | New York, USA | AAAR9499. Columbia University Irving Medical Center - 18/06/2018 |

**References:**

1. Le Gall JR, Lemeshow S, Saulnier F: A new Simplified Acute Physiology Score (SAPS II) based on a European/North American multicenter study. *JAMA* 1993; 270:2957–2963

2. Vincent JL, Moreno R, Takala J, et al.: The SOFA (Sepsis-related Organ Failure Assessment) score to describe organ dysfunction/failure. On behalf of the Working Group on Sepsis-Related Problems of the European Society of Intensive Care Medicine. *Intensive Care Med* 1996; 22:707–710

3. Schmidt M, Bailey M, Sheldrake J, et al.: Predicting survival after extracorporeal membrane oxygenation for severe acute respiratory failure. The Respiratory Extracorporeal Membrane Oxygenation Survival Prediction (RESP) score. *American journal of respiratory and critical care medicine* 2014; 189:1374–82
