# Supplementary material for: Tracheostomy management in patients with severe acute respiratory distress syndrome receiving extracorporeal membrane oxygenation: an International Multicenter Retrospective Study
Source: Crit Care. 2021 Jul 7;25:238. doi: 10.1186/s13054-021-03649-8 (PMC8261805; doi:10.1186/s13054-021-03649-8)
Supplement: Supplementary file 2 — Additional file 2. Characteristics of the four international ECMO centers and their tracheostomy management. [file 13054_2021_3649_MOESM2_ESM.docx]

**Additional file 2. Characteristics of the four international ECMO centers and their tracheostomy management**

|  | **Regensburg, Germany** | **New-York, USA** | **Palermo, Italy** | **Paris, France** |
| --- | --- | --- | --- | --- |
| **Characteristics of the center** |  |  |  |  |
| University hospital | Yes | Yes | No | Yes |
| Number of hospital beds | 893 | 700 | 110 | 1,200 |
| Number of ICU beds in the included ICUs | 16 | 24 | 20 | 26 |
| Number of ICU admissions per year in the included ICUs | 700 | 1,200 | 960 | 1,100 |
| Number of adult patients ECMO-treated primarily for respiratory indications in the last calendar year | 60 | 57 | 25 | 65 |
| Number of adult patients ECMO–treated primarily for cardiac indications in the last calendar year | 140 | 1 | 32 | 240 |
| Patient-to-nurse ratio | 2/1 | 2/1 | 2/1 | 2/1 |
| Patient-to-nurse ratio for patients on ECMO | 2/1 | 1/1 | 1/1 | 2/1 |
| Mobile ECMO team | Yes | Yes | Yes | Yes |
| VV cannulation performed by surgeons | No | Yes | No | Yes |
| VV cannulation performed by ICU physicians | Yes | No | Yes | No |
| **Tracheostomy management** |  |  |  |  |
| Primary tracheostomy procedure | Percutaneous | Percutaneous | Percutaneous | Percutaneous |
| Delay to stop heparin before the procedure | 4 | 4 | 4 | 4 |
| Delay to restart heparin after the procedure | 2 | 2 | 2 | 2 |
| Minimum platelets count to perform a tracheostomy: |  |  |  |  |
| - during ECMO, /mm^3^ | 30,000 | 50,000 | 50,000 | 80,000 |
| - without ECMO, /mm^3^ | 50,000 | 50,000 | 50,000 | 80,000 |
| Minimum hemoglobin to perform a tracheostomy: | level |  |  |  |
| - during ECMO, g/dL | 7 | 7 | 7 | 7 |
| - without ECMO, g/dL | 8 | 7 | 7 | 7 |
| Minimum fibrinogen level to perform a tracheostomy: |  |  |  |  |
| - during ECMO, mg/dL | 150 | 100 | - | 200 |
| - without ECMO, mg/dL | 150 | 100 | - | 200 |
| **Number of time Richmond Agitation Sedation Scale reported per day** | 6 | 2 | 3 | 6 |

*ECMO, extracorporeal membrane oxygenation; ICU, intensive care unit, VV, venovenous*
