# Supplementary material for: Tracheostomy management in patients with severe acute respiratory distress syndrome receiving extracorporeal membrane oxygenation: an International Multicenter Retrospective Study
Source: Crit Care. 2021 Jul 7;25:238. doi: 10.1186/s13054-021-03649-8 (PMC8261805; doi:10.1186/s13054-021-03649-8)
Supplement: Supplementary file 3 — Additional file 3. ECMO-related complications according to the timing of tracheostomy. [file 13054_2021_3649_MOESM3_ESM.docx]

**Additional file 3: ECMO-related complications according to the timing of tracheostomy.**

|  | **All patients (n=1,168)** | **During-ECMO tracheostomy (n=348)** | **Post-ECMO tracheostomy (n=182)** | **No tracheostomy (n=638)** | **Global**  ***P-*value** |
| --- | --- | --- | --- | --- | --- |
| Stroke |  |  |  |  |  |
| Ischemic | 27 (2) | 10 (3) | 5 (3) | 12 (2) | 0.51 |
| Hemorrhagic | 38 (3) | 19 (5) | 5 (3) | 14 (2)^#^ | 0.02 |
| At least one major bleeding event | 193 (16) | 107 (31) | 25 (14) ^#^ | 61 (10) ^#^ | <0.01 |
| Hemothorax | 45 (4) | 32 (9) | 10 (5) | 3 (1) ^#*^ | <0.01 |
| Ear-Nose-Throat | 92 (8) | 54 (15) | 16 (9) ^#^ | 22 (3) ^#*^ | <0.01 |
| Gastrointestinal | 66 (6) | 42 (12) | 11 (6) ^#^ | 13 (2) ^#*^ | <0.01 |
| ECMO cannula | 82 (7) | 47 (13) | 14 (8) | 21 (3) ^#*^ | <0.01 |
| Intracranial | 54 (5) | 23 (7) | 16 (9) | 15 (2) ^#*^ | <0.01 |
| Transfused RBC units | 6.9 ± 10.0 | 11.4 ± 13.2 | 7.1 ± 9.6^#^ | 4.5 ± 6.9^#*^ | <0.01 |
| Transfused RBC units per ECMO day | 0.68 ± 1.40 | 0.54 ± 0.69 | 0.80 ± 1.12 | 0.72 ± 1.72 | 0.07 |

*ECMO, extracorporeal membrane oxygenation ; RBC, red blood cells*

*#, p-value<0.05 vs “tracheostomy during ECMO”; *, p-value<0.05 vs “post-ECMO tracheostomy”*
