# Supplementary material for: Tracheostomy management in patients with severe acute respiratory distress syndrome receiving extracorporeal membrane oxygenation: an International Multicenter Retrospective Study
Source: Crit Care. 2021 Jul 7;25:238. doi: 10.1186/s13054-021-03649-8 (PMC8261805; doi:10.1186/s13054-021-03649-8)
Supplement: Supplementary file 4 — Additional file 4. Pre-ECMO variables associated with the decision to perform a tracheostomy (i.e., during or after ECMO) in severe ARDS patients. [file 13054_2021_3649_MOESM4_ESM.docx]

**Additional file 4. Pre-ECMO variables associated with the decision to perform a tracheostomy (i.e. during or after ECMO) in severe ARDS patients.**

| **Variable** | **HR (95%CI)** | ***P* value** |
| --- | --- | --- |
| Columbia University, New York, United States of America | 2.11 (1.28-3.51) | <0.01 |
| University Hospital Regensburg, Regensburg, Germany | 1.78 (1.14-2.82) | 0.01 |
| Immunocompromised status | 1.58 (1.06-2.37) | 0.02 |
| SOFA score at cannulation (respiratory and neurological components excluded) | 0.85 (0.81-0.90) | <0.01 |
| Surgery within 7 days before ARDS onset | 2.41 (1.70-3.43) | <0.01 |
| Prone position before ECMO | 2.21 (1.44-3.43) | <0.01 |
| Pneumothorax before ECMO | 5.35 (3.16-9.42) | <0.01 |
| Extra pulmonary infection | 1.96 (1.17-3.30) | 0.01 |
| Corticosteroids before ECMO | 2.37 (1.59-3.56) | <0.01 |
| Known restrictive lung disease | 6.06 (1.53-40.6) | 0.02 |
| Bacterial pneumonia | 1.71 (1.25-2.35) | <0.01 |
| Viral pneumonia | 3.54 (2.31-5.46) | <0.01 |
| Pancreatitis | 4.95 (1.18-25.9) | 0.04 |

*ARDS, acute respiratory distress syndrome; CI, confidence interval; ECMO, extracorporeal membrane oxygenation; HR, hazard ratio; SOFA, Sequential Organ Failure Assessment*

*Area under the curve of the model 0.70 (0.65-0.75)*
