# Supplementary material for: Tracheostomy management in patients with severe acute respiratory distress syndrome receiving extracorporeal membrane oxygenation: an International Multicenter Retrospective Study
Source: Crit Care. 2021 Jul 7;25:238. doi: 10.1186/s13054-021-03649-8 (PMC8261805; doi:10.1186/s13054-021-03649-8)
Supplement: Supplementary file 5 — Additional file 5 Impact of tracheostomy on the unfractionated heparin dose and the packed red blood cell transfusion according to the timing of tracheostomy. [file 13054_2021_3649_MOESM5_ESM.docx]

# Additional file 5: Impact of tracheostomy on the unfractionated heparin dose and the packed red blood cell transfusion according to the timing of tracheostomy


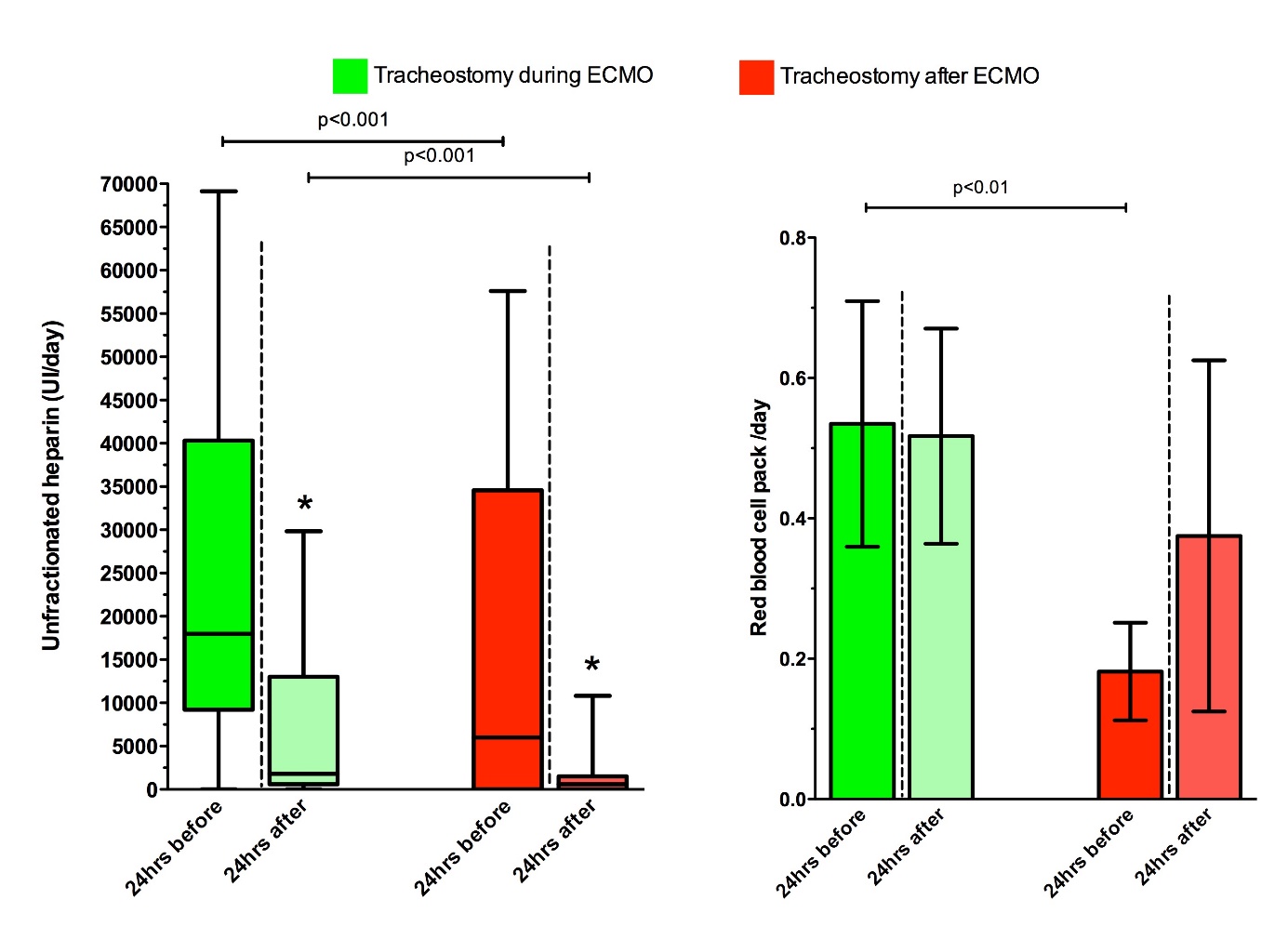


*ECMO extracorporeal membrane oxygantion, hrs, hours*
